# Day-21 gut microbiota community state types are associated with bronchopulmonary dysplasia classification in preterm infants: a pilot shotgun metagenomic study

**DOI:** 10.3389/fmicb.2026.1835952

**Published:** 2026-07-06

**Authors:** Wenjun Xiong, Xudong Yan, Hui Guo, Boshi Yu, Jie Qi, Haitao Li, Zhipeng Zeng, Yong Dai, Zhangbin Yu, Donge Tang

**Affiliations:** 1 Clinical Medical Research Center, Shenzhen People's Hospital (The First Affiliated Hospital, Southern University of Science and Technology, The Second Clinical Medical College, Jinan University), Shenzhen, China; 2 Forensic Evidence Laboratory, Shenzhen People's Hospital (The First Affiliated Hospital, Southern University of Science and Technology, The Second Clinical Medical College, Jinan University), Shenzhen, China; 3 Department of Neonatology, Shenzhen People's Hospital (The First Affiliated Hospital, Southern University of Science and Technology, The Second Clinical Medical College, Jinan University), Shenzhen, China; 4 School of Medicine, Anhui University of Science & Technology, Huainan, China

**Keywords:** bronchopulmonary dysplasia (BPD), clinical risk assessment, gut microbiadysbiosis, preterm infants, state types

## Abstract

**Introduction:**

Bronchopulmonary dysplasia (BPD) is a major complication in preterm infants, and its clinical classification remains strongly influenced by gestational maturity and the evolving respiratory course. In this exploratory study, we investigated whether early-life gut microbiota configurations at postnatal day 21 are associated with subsequent formal BPD classification at 36 weeks postmenstrual age and whether they provide ecological information relevant to preterm infant microbiome development.

**Methods:**

In a prospective cohort of 23 preterm infants with gestational age <32 weeks or birth weight <1,500 g, shotgun metagenomic sequencing of day-21 fecal samples was performed. Community state types (CSTs) were identified using unsupervised clustering, and their taxonomic, functional, and exploratory discrimination patterns were assessed in relation to subsequent BPD classification.

**Results:**

Two CSTs were identified. CST1 was dominated by commensal taxa and exhibited functional enrichment in metabolic homeostasis pathways. CST2 was characterized by pathobionts, fragmented taxon–pathway association networks, and enrichment in virulence-related pathways. BPD was observed in 1 of 11 CST1 infants and 7 of 12 CST2 infants (9.1% vs. 58.3%; two-sided Fisher’s exact test, p = 0.027), although this association was based on very small cell counts. In exploratory discrimination analysis, a model combining CST status with gestational age showed an apparent AUC of 0.892; however, leave-one-out cross-validation yielded a lower AUC of 0.800, indicating likely optimism in the apparent model performance.

**Discussion:**

These preliminary, observational findings suggest that day-21 gut microbiota profiles and CST classification may provide ecological information associated with formal BPD classification. However, this analysis should be interpreted as exploratory discrimination rather than validation of a clinically useful prediction model. Establishing causality or clinical utility requires validation in larger cohorts that systematically track longitudinal confounders such as gestational age, feeding mode, antibiotics, and probiotics.

## Highlights

- Early-life gut microbiota at day 21 in preterm infants exhibits two distinct ecological phenotypes (CST1 and CST2), characterized by divergent taxonomic, functional, and network-level features.
- CST typing was associated with formal BPD classification at 36 weeks postmenstrual age, but this association was strongly intertwined with gestational age and should be interpreted as exploratory.
- These exploratory findings support further investigation of microbiota-based ecological phenotypes in larger longitudinal cohorts with systematic capture of antibiotic exposure, feeding mode, probiotic use, and clinical severity.

## 1 Introduction

Bronchopulmonary dysplasia (BPD) remains one of the most common and severe complications in preterm infants, leading to lifelong respiratory morbidity and increased mortality (Jensen et al., 2019; Stoll et al., 2015). Clinical risk assessment remains important for individualized management, yet current models rely mainly on gestational age, birth weight, and respiratory support history and therefore have limited individual-level precision. Existing clinical indicators often reflect an evolving respiratory trajectory after weeks of respiratory support, oxygen exposure, and intensive care treatment before formal BPD classification at 36 weeks postmenstrual age (Peng et al., 2022).

Growing evidence implicates the gut microbiota in neonatal immune maturation and lung development via the gut–lung axis (Özçam and Lynch, 2024; Sanidad and Zeng, 2020; Tirone et al., 2019). Early-life dysbiosis can disrupt intestinal barrier integrity, promote systemic inflammation, and exacerbate pulmonary injury through pathogen-associated molecular pattern (PAMP) translocation and reduced short-chain fatty acid (SCFA)–mediated immunoregulation (Ney et al., 2023). Importantly, recent work has expanded this paradigm beyond bacteria: Willis et al. demonstrated that gut fungal dysbiosis not only predicts but causally drives BPD susceptibility in very low birthweight infants, supported by fecal microbiota transplant experiments in antibiotic-pseudo humanized mice (Willis et al., 2023). Multi-omics studies further link gut microbiota composition and microbiota-derived metabolites to BPD severity in preterm infants (Ryan et al., 2019). Together, these findings underscore the need to move beyond descriptive taxonomic analyses and toward ecological characterizations that capture emergent properties such as network connectivity, metabolic redundancy, and inflammatory potential (Tannock, 2024).

Despite these advances, two major knowledge gaps remain: (1) no consensus exists on a developmentally meaningful and clinically feasible time point for gut microbiome sampling in preterm infants (Healy et al., 2022), and (2) prior studies often focus on individual taxa or diversity metrics rather than community-level ecological states (Groer et al., 2014; Pammi et al., 2017). Postnatal day 21 represents a pragmatic sampling milestone in the NICU setting—the transition from parenteral to enteral feeding is often underway, though its pace varies with gestational age and clinical stability (Oddie et al., 2021), early microbial volatility begins to stabilize (Thänert et al., 2024), and for infants born at the lowest gestational ages, the lung may still be in the saccular stage of development, although more mature infants may have progressed further (Burri, 2006). It should be noted that BPD is a developmental lung disease whose pathological trajectory begins at birth: indicators of likely BPD, including prolonged mechanical ventilation and oxygen dependence, are often evident well before the formal diagnostic threshold at 36 weeks postmenstrual age. Day 21 should therefore not be construed as preceding disease onset *per se*, but rather as a time point at which gut microbiota sampling is feasible and community structure may already reflect the cumulative effects of prematurity, respiratory support, and antibiotic exposure. We reasoned that gut communities at this point may reflect early ecological states associated with subsequent respiratory outcomes. We conceptualized these community types as microbiome-derived ecological phenotypes, capturing emergent taxonomic, metabolic, and interaction-level features with potential relevance for host development.

Therefore, we hypothesized that distinct community state types (CSTs) are present at day 21 and are associated with subsequent formal BPD classification. To test this hypothesis, we conducted shotgun metagenomic sequencing of day-21 fecal samples from preterm infants and adjudicated BPD status at 36 weeks postmenstrual age. We defined CSTs using unsupervised clustering (Ravel et al., 2011), performed exploratory discrimination analyses, characterized their taxonomic, functional, and network-level features, and assessed associations with clinical and immunological parameters. Our goal was to evaluate whether microbiome-derived ecological phenotypes (Zhang et al., 2022) could provide exploratory stratification information and generate hypotheses for future longitudinal studies.

## 2 Materials and methods

### 2.1 Ethics and participant enrollment

This prospective cohort study was conducted in the NICU of Shenzhen People’s Hospital, with ethics approval obtained from the hospital’s Ethics Committee (Approval No. LL-KY-2022494-02), in accordance with the Declaration of Helsinki. Written informed consent was obtained from the parents or legal guardians of all participants.

Formal enrollment, informed consent acquisition, and sample collection were conducted between March 13, 2023, and March 12, 2024. Eligible participants were preterm neonates (gestational age < 32 weeks or birth weight < 1,500 g) admitted to the NICU within 24 h of birth. Exclusion criteria included major congenital malformations, chromosomal abnormalities, severe gastrointestinal diseases, death before 36 weeks’ postmenstrual age, or absence of a fecal sample on day 21.

**Figure 1:**
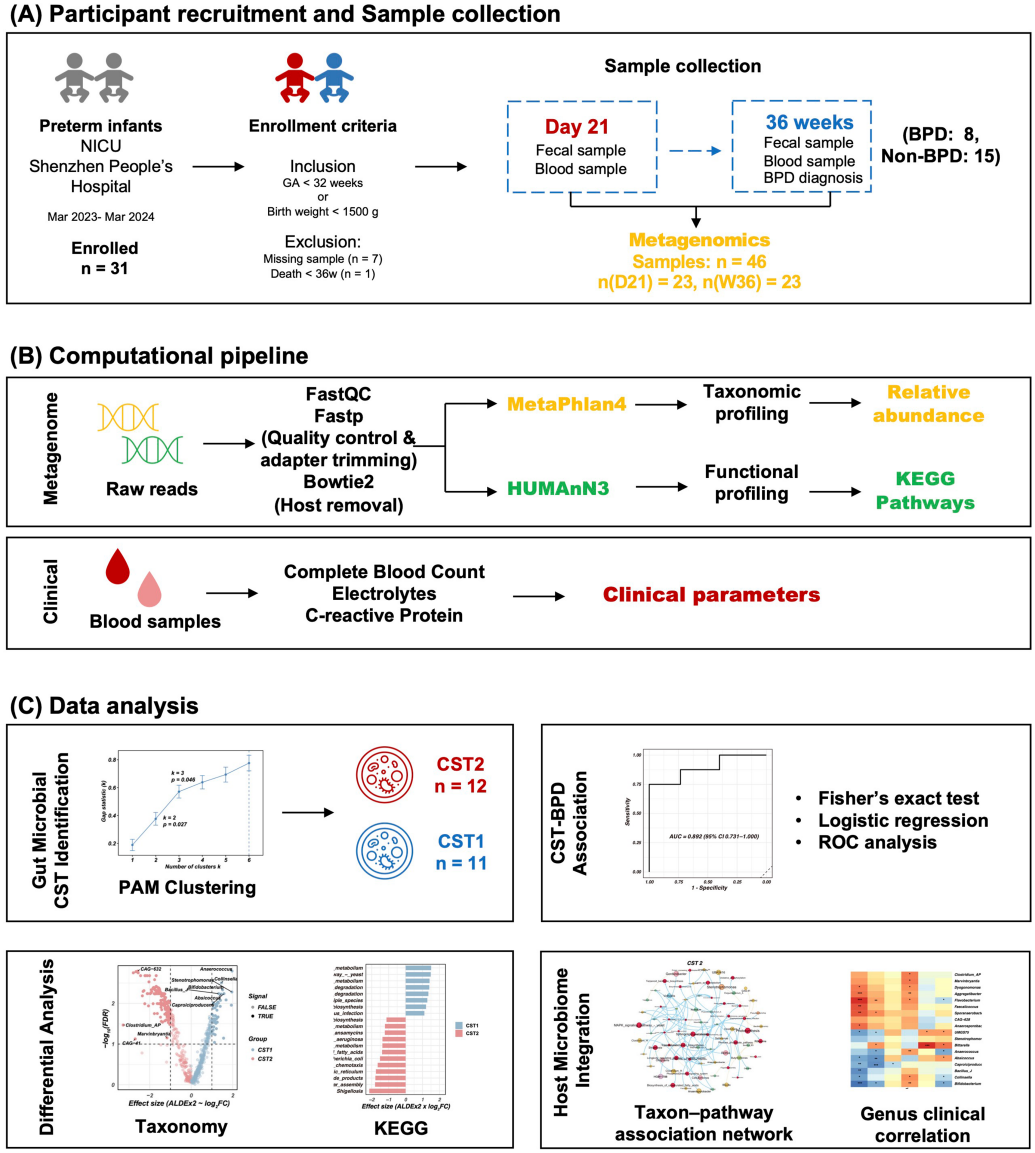
Workflow for gut microbiota analysis and exploratory BPD association assessment in preterm infants. **(A)** Participant recruitment and sample collection. Preterm infants (GA < 32 weeks or birth weight <1,500 *g*) were enrolled from the NICU of Shenzhen People’s Hospital (March 2023–Mar 2024) of 31 enrolled infants, 23 were included in the final analysis (8 BPD, 15 non-BPD). Fecal and blood samples were collected at day 21 and at 36 weeks postmenstrual age. Metagenomic sequencing was performed at both time points, with CST identification and BPD association analyses restricted to Day 21 samples. Week 36 samples were used as ecological references to assess microbial maturation. **(B)** Computational pipeline. Metagenomic reads underwent quality control and adapter trimming (FastQC, fastp) and host removal (Bowtie2), followed by taxonomic (MetaPhlAn4) and functional profiling (HUMAnN3). Clinical parameters were extracted from blood samples. **(C)** Data analysis. CSTs were identified using PAM clustering. CST–BPD associations were evaluated using Fisher’s exact test, exploratory logistic regression, and ROC analysis with leave-one-out cross-validation. Differential abundance (ALDEx2), taxon–pathway association network, and genus–clinical correlation analyses were performed.

Of 31 eligible infants, 8 were excluded (7 with missing day-21 samples, 1 deceased), leaving 23 infants for analysis. BPD was diagnosed in 8 infants per 2019 NICHD criteria; 15 infants without BPD served as controls (Figure 1A). Key clinical variables were defined as follows. Neonatal respiratory distress syndrome (NRDS) was diagnosed based on clinical presentation and/or chest radiographic findings consistent with surfactant deficiency. Patent ductus arteriosus (PDA) was diagnosed by echocardiographic confirmation of ductal patency. Premature rupture of membranes (PROM) was defined as rupture of membranes ≥18 h before delivery. Antenatal corticosteroids were recorded as administered if any dose of antenatal corticosteroid therapy was given prior to delivery, regardless of course completeness. Maternal antibiotic treatment refers to any antibiotic administration during the antepartum or intrapartum period, regardless of indication or duration.

### 2.2 Sample collection

Fecal samples were collected on postnatal day 21 (±3 days) and at 36 weeks’ postmenstrual age (±3 days). Shotgun metagenomic sequencing was performed for both time points (*n* = 23 each), yielding a total of 46 datasets. Samples were collected under sterile conditions and immediately stored at −80 °C until analysis. Peripheral blood was obtained at the same time points for complete blood counts, C-reactive protein (CRP), and electrolyte measurements. Available clinical records showed that no systemic antibiotics were administered within 14 days before the day-21 stool sampling and that no infant received probiotic supplementation during the study period. Detailed quantitative feeding-mode proportions at day 21 were unavailable for formal adjustment.

### 2.3 Shotgun metagenomic sequencing and taxonomic and functional annotation

Total fecal DNA was extracted using the Qiagen QIAamp DNA Stool Mini Kit. Libraries were prepared with the Illumina Nextera XT DNA Library Preparation Kit. Sequencing was performed on an Illumina NovaSeq 6,000 platform to generate high-quality 150 bp paired end reads (PE150). Raw reads were assessed for quality using FastQC. Adapter trimming and quality filtering were performed using fastp (Chen et al., 2018). Human reads were removed using Bowtie2 (Langmead and Salzberg, 2012). Community composition was profiled with MetaPhlAn4 (Blanco-Míguez et al., 2023), and functional profiling was performed with HUMAnN3 (Beghini et al., 2021). Reads were mapped to UniRef90 protein families and then regrouped to KEGG Orthology and pathways using the KEGG database (Kanehisa and Goto, 2000). The final outputs were genus-level relative abundance tables and KEGG pathway abundances for each sample, with downstream analyses focused on day-21 data (Figure 1B).

### 2.4 Community state typing of preterm infant gut microbiota

**Figure 2:**
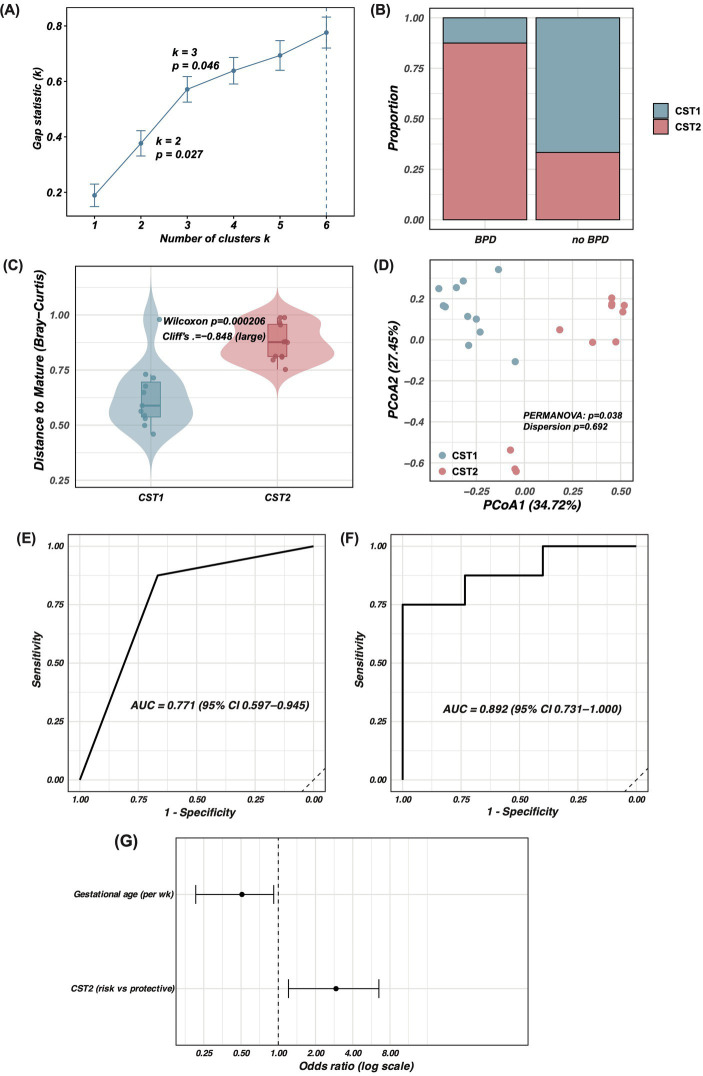
Gut microbial community state types at day 21 are associated with BPD classification in preterm infants. **(A)** Gap statistical analysis for determining the optimal number of clusters. The gut microbiota at postnatal day 21 was partitioned into a two-CST structure (*k* = 2, *p* = 0.027), with *k* = 3 also showing statistical support (*p* = 0.046), indicating that the clustering structure should be interpreted cautiously in this small cohort. Error bars represent standard error from bootstrap resampling. **(B)** Distribution of BPD outcomes across day-21 CSTs. BPD was observed in 1 of 11 infants assigned to CST1 (9.1%) and in 7 of 12 infants assigned to CST2 (58.3%) (two-sided Fisher’s exact test, *p* = 0.027). **(C)** Violin plot comparing Bray–Curtis dissimilarity to the internal week-36 non-BPD reference between CST1 and CST2. CST1 samples were significantly closer to this internal reference than CST2 samples (Wilcoxon *p* < 0.001, Cliff’s *δ* = −0.848). Because the reference was derived from non-BPD infants within the same cohort, this analysis should be interpreted as corroborative rather than as an independent maturation benchmark. **(D)** Principal coordinates analysis (PCoA) based on Bray–Curtis dissimilarity demonstrating distinct separation between CST1 (blue) and CST2 (red) communities. PERMANOVA confirmed significant differences in community composition (*p* = 0.038), while homogeneity of dispersion was not violated (*p* = 0.692). The first two principal coordinates explained 62.17% of the total variance. **(E)** Exploratory ROC curve for BPD classification using CST2 status alone, showing an apparent AUC of 0.771. **(F)** Exploratory ROC curve for a model incorporating CST2 status and gestational age, showing an apparent in-sample AUC of 0.892. Leave-one-out cross-validation yielded a lower AUC of 0.800, indicating likely optimism in the apparent estimate. **(G)** Exploratory adjusted logistic regression including CST2 status and gestational age. Effect estimates were imprecise because only eight BPD events were available; therefore, this model was used for sensitivity assessment rather than for drawing conclusions about independent associations.

Based on genus-level relative abundances from day-21 samples (*n* = 23), we computed Bray–Curtis distance matrices (Bray and Curtis, 1957) and visualized compositional variation using principal coordinates analysis. Clustering was performed using the partitioning around medoids (PAM) algorithm (Modak, 2023) on the Bray–Curtis matrix, with cluster validity assessed by silhouette coefficients (Rousseeuw, 1987) across *k* = 2–5. The optimal solution was *k* = 2 (silhouette score = 0.72), supported by the gap statistic (*p* = 0.027) (Tibshirani et al., 2001) and ordination patterns (Figure 2A). Samples were thus classified into two community state types, CST1 and CST2 (Figure 1C). Cluster stability was assessed via bootstrap resampling (1,000 iterations), yielding 89.3% consistency.

To assess ecological maturation, we used the 36-week PMA fecal samples of the 15 non-BPD infants within our cohort as an internal reference representing a mature gut configuration. For each day-21 sample, Bray–Curtis distances to this reference group were computed as the mean distance to each of the 15 reference samples, and group-level comparisons between CST1 and CST2 were performed using the Wilcoxon rank-sum test (Figure 2C).

### 2.5 Differential taxa and marker taxa

To identify marker genera that discriminate CST1 from CST2, we first applied ALDEx2 (v1.28) with the Welch’s t-test option (mc.samples = 128) to assess differential abundance based on centered log-ratio transformed counts. We additionally calculated the mean relative abundance and detection rate for each genus in the two CSTs, where detection rate was defined as the proportion of samples in which a genus was detected. Between-CST differences in abundance were tested with the Mann–Whitney test (Mann and Whitney, 1947) and differences in detection rates with Fisher’s exact test (Fisher, 1949). *p-*values were adjusted by the Benjamini–Hochberg method (Benjamini and Hochberg, 1995). A genus was defined as a marker if both abundance and detection rate differed significantly at FDR < 0.05 (Xu et al., 2022) and if it was a dominant genus in one CST (Figure 1C).

### 2.6 Differential metabolic functions

We quantified metagenomic functional abundances using KEGG annotations (Levels 1–3), focusing on metabolic and inflammation-related pathways at Level 3, including lipopolysaccharide biosynthesis, sphingolipid metabolism, vitamin biosynthesis, and xenobiotic degradation. We summarized Level 2 categories in CST1 and CST2, identified significantly enriched functions, and further examined selected Level 3 pathways. Group comparisons were performed using Mann–Whitney tests. Given the high dimensionality (1,243 pathways) and the exploratory nature of enrichment analysis, significance was set at FDR < 0.1, aligned with prior microbiome studies in preterm infants (Figure 1C) (Xu et al., 2022).

### 2.7 Microbe–function association networks

To explore CST-specific taxon–function relationships, we constructed genus–function bipartite networks within each CST based on Spearman correlations (|*ρ*| > 0.8, *p* < 0.05). Edges linked genera and KEGG pathways, and genus–genus co-function networks were derived by projecting nodes sharing multiple functional links. Edge weights reflected either shared function counts or mean correlation strengths. Network topology—including node degree, density, and clustering coefficients—was summarized descriptively to visualize CST-specific taxon–function co-variation (Figure 1C).

### 2.8 Statistical analysis

Unless otherwise specified, analyses were conducted in R v4.1.0. Given the small sample size, continuous clinical variables were summarized as median with interquartile range (IQR) and compared using the Mann–Whitney U test. Categorical variables were summarized as counts and percentages and compared using Fisher’s exact test. Correlations were assessed using Spearman’s rho. Multiple testing was controlled using the Benjamini–Hochberg method where applicable. Two-sided p < 0.05 or FDR < 0.05 indicated statistical significance. Figures were prepared with ggplot2. Alpha diversity was calculated from species-level absolute abundance profiles using Shannon diversity, Simpson diversity, observed species richness, and Chao1 richness. Sequencing-depth and read-processing metrics, including raw reads, clean reads, host-removed reads, host rate, Q30, GC content, and clean data rate, were summarized for each sample and compared between CST groups. For exploratory discrimination analysis, apparent AUC values were calculated for CST2 status alone, gestational age alone, and CST2 plus gestational age. Leave-one-out cross-validation was additionally performed to estimate optimism in model performance. Genus–clinical marker correlations were assessed using Spearman correlation, and Benjamini–Hochberg correction was applied across all displayed correlations. To evaluate whether the Bifidobacterium–calcium association was explained by gestational age, partial Spearman correlation was performed using rank residuals after adjustment for gestational age.

## 3 Results

### 3.1 Baseline data

Infants in the BPD group had lower gestational age than non-BPD infants [26.5 (24.8–27.2) weeks vs. 30.0 (28.5–31.0) weeks, *p* = 0.002], lower birth weight [840 (738–1,080) g vs. 1,180 (1005–1,325) g, *p* = 0.018], and lower Apgar scores at both 1 and 5 min. Infants in the BPD group also required longer mechanical ventilation, longer oxygen therapy, longer parenteral nutrition, and longer hospitalization. No significant differences were observed in sex, NRDS, PDA, or corrected gestational age at discharge (Table 1).

**Table 1:** Baseline characteristics of infants in the BPD and non-BPD groups.

**Characteristic**	**BPD (*n* = 8)**	**Non-BPD (*n* = 15)**	***p***
Birth weight (g)	840 (738–1,080)	1,180 (1005–1,325)	0.018
Gestational age (weeks)	26.5 (24.8–27.2)	30.0 (28.5–31.0)	0.002
Apgar score at 1 min	8.0 (5.0–8.2)	10.0 (8.5–10.0)	0.017
Apgar score at 5 min	9.0 (8.8–10.0)	10.0 (10.0–10.0)	0.013
Male sex, *n* (%)	5 (62.5%)	10 (66.7%)	1.000
NRDS, *n* (%)	6 (75%)	10 (66.7%)	1.000
Patent ductus arteriosus (PDA) *n* (%)	3 (37.5%)	3 (20.0%)	0.614
Duration of mechanical ventilation (days)	56.0 (36.5–71.2)	9.0 (5.0–13.5)	<0.001
Duration of oxygen therapy (days)	65.0 (44.0–96.5)	9.0 (6.0–13.0)	<0.001
Length of hospital stay (days)	87.0 (72.0–112.8)	52.0 (37.5–60.0)	0.002
Corrected GA at discharge (weeks)	38.0 (37.2–39.5)	37.0 (36.8–37.2)	0.092
Duration of parenteral nutrition (days)	33.5 (29.2–41.5)	23.0 (16.5–32.0)	0.030

Continuous variables are presented as median (IQR); categorical variables as n (%). *p*-values from Mann–Whitney *U* test (continuous) or Fisher’s exact test (categorical). Duration of oxygen therapy includes all supplemental oxygen support excluding days on invasive mechanical ventilation.

Regarding maternal and prenatal characteristics, mothers in the BPD group were older than those in the non-BPD group [36.0 (33.0–36.2) years vs. 31.0 (28.5–32.5) years, *p* = 0.023]. Cesarean section, antenatal steroid exposure, PROM, and maternal antibiotic treatment did not differ significantly between groups (Table 2).

**Table 2:** Maternal and prenatal characteristics.

**Characteristic**	**BPD (*n* = 8)**	**Non-BPD (*n* = 15)**	***p***
Maternal age (years)	36.0 (33.0–36.2)	31.0 (28.5–32.5)	0.023
Cesarean section, *n* (%)	6 (75.0%)	12 (80.0%)	1.000
Antenatal steroids, *n* (%)	7 (87.5%)	13 (86.7%)	1.000
PROM, *n* (%)	1 (12.5%)	2 (13.3%)	1.000
Maternal antibiotic treatment, *n* (%)	6 (85.7%)	12 (80.0%)	1.000

Continuous variables are presented as median (IQR); categorical variables as n (%). *p*-values from Mann–Whitney *U* test (continuous) or Fisher’s exact test (categorical).

### 3.2 Day-21 gut microbiota CSTs are associated with formal BPD classification

We performed shotgun metagenomic sequencing on fecal samples collected at postnatal day 21 and adjudicated BPD status at 36 weeks postmenstrual age. Unsupervised clustering identified a two-CST structure in this pilot dataset (Figure 2A). The *k* = 2 partition was supported by gap statistics (*p* = 0.027), silhouette analysis (score = 0.72 at *k* = 2), and bootstrap resampling consistency (89.3%), although the small sample size limits the certainty of this clustering solution. Eleven infants were assigned to CST1 and twelve to CST2.

These CSTs were associated with subsequent formal BPD classification. Among infants assigned to CST1, 9.1% (1/11) developed BPD, compared with 58.3% (7/12) of infants assigned to CST2 (two-sided Fisher’s exact test, *p* = 0.027; OR = 14.00, 95% CI 1.33–147.38) (Figure 2B and Supplementary Table S2). However, the confidence interval was wide, and the association was sensitive to the classification of one infant, supporting a cautious, hypothesis-generating interpretation. Ecologically, Bray–Curtis distance analysis showed that CST1 samples were closer to the internal week-36 non-BPD reference than were CST2 samples (Wilcoxon *p* < 0.001, Cliff’s *δ* = −0.848) (Figure 2C). Because this reference was derived from non-BPD infants within the same cohort, this analysis should be interpreted as corroborative rather than as independent evidence of delayed ecological maturation. All ordination and distance analyses were performed on day-21 samples (*n* = 23) (PERMANOVA *R*^2^ = 0.18, *p =* 0.038; dispersion *p* = 0.692) (Figure 2D).

In exploratory ROC analysis, CST2 status alone showed an apparent AUC of 0.771, whereas gestational age alone showed an apparent AUC of 0.875. Combining CST2 status with gestational age yielded an apparent AUC of 0.892. However, leave-one-out cross-validation reduced the AUC of the combined model to 0.800, and the CST2-only LOOCV AUC was 0.583 (Supplementary Figure S3C). These results indicate optimism in the apparent model performance and underscore the need for external validation. The adjusted logistic model was retained as an exploratory sensitivity analysis rather than as evidence of independent predictive value (Figures 2E–G). Because gestational age differed between BPD and non-BPD infants, we formally assessed gestational-age imbalance across CST groups. Infants assigned to CST2 had significantly lower gestational age than those assigned to CST1 [median 28.21 vs. 31.71 weeks, *p* = 0.0015] and lower birth weight [median 1,080 vs. 1,350 g, *p* = 0.011]. Consistently, BPD infants had lower gestational age than non-BPD infants [median 27.28 vs. 30.86 weeks, *p* = 0.004] (Supplementary Figure S3 and Supplementary Table S2). These findings indicate that gestational age is an important confounder of the CST–BPD association.

### 3.3 Distinct taxonomic signatures define CST1 as commensal-driven and CST2 as pathobiont-dominated

To provide standard ecological context for the CST classification, we further evaluated alpha diversity at day 21. Compared with CST1, CST2 showed significantly lower Shannon diversity, Simpson diversity, observed species richness, and Chao1 richness (all *p* ≤ 0.027; Supplementary Figure S1A and Supplementary Table S3). When infants were grouped by BPD status, BPD infants showed significantly lower observed species richness and Chao1 richness (both *p* = 0.002), whereas Shannon and Simpson diversity showed non-significant downward trends (Supplementary Figure S1B and Supplementary Table S3). To evaluate whether CST-associated differences were driven by sequencing-depth imbalance, we compared read-processing metrics between CST1 and CST2. Raw reads, clean reads, host-removed reads, host rate, Q30, GC content, and clean data rate did not differ significantly between CST groups (all *p* > 0.05; Supplementary Figure S2A and Supplementary Table S1), suggesting that the observed CST-associated ecological differences were unlikely to be explained by sequencing-depth variation. Rarefaction curves showed comparable richness saturation patterns across day-21 samples (Supplementary Figure S2B).

**Figure 3:**
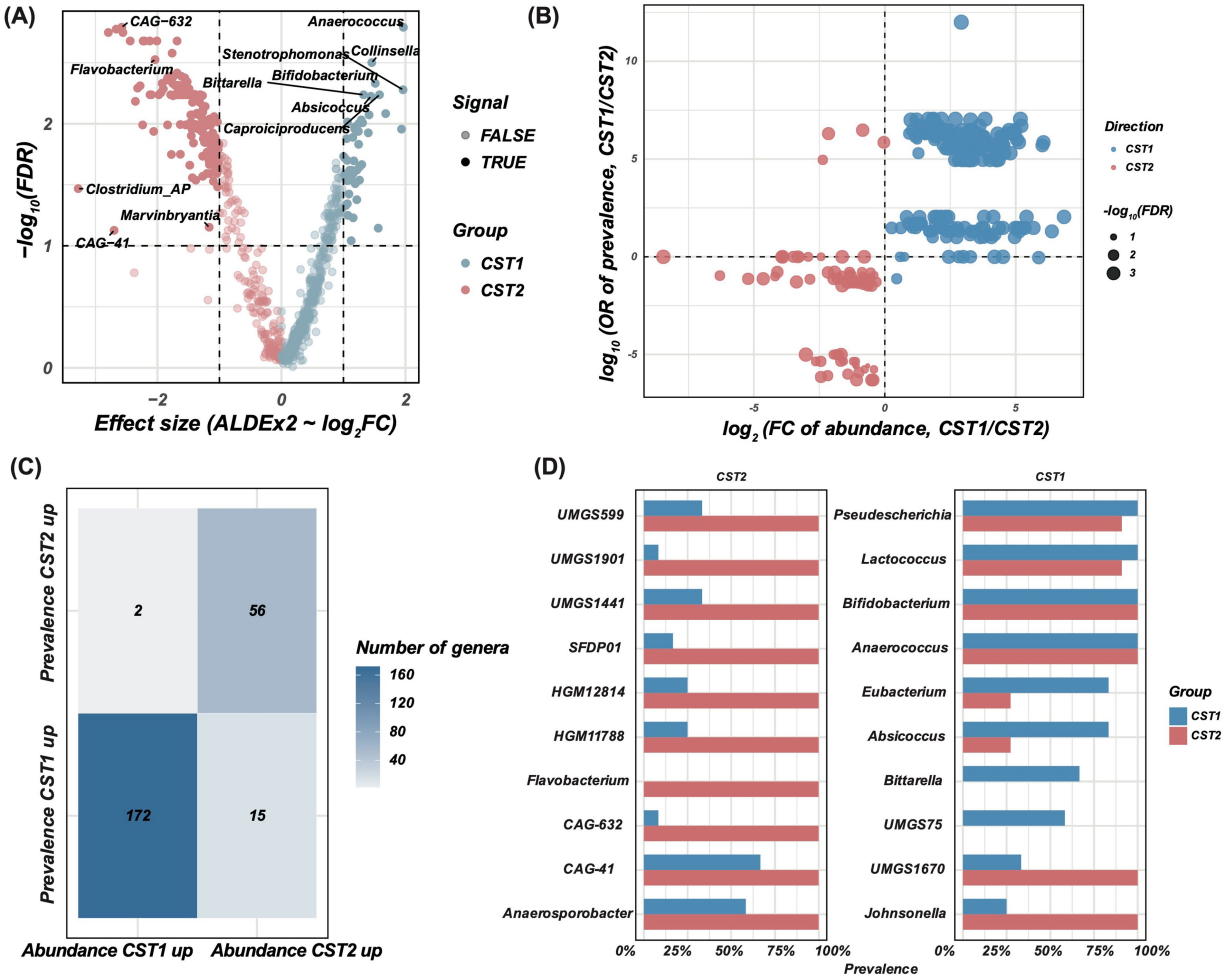
Taxonomic composition and differential abundance analysis between CST1 and CST2. **(A)** Volcano plot showing differentially abundant genera between CST1 and CST2 identified by ALDEx2 analysis. The *x*-axis represents effect size (log₂ fold change), and the *y*-axis represents statistical significance (−log₁₀ FDR). CST1-enriched genera (blue) included *Bifidobacterium*, *Collinsella*, *Anaerococcus*, *Absicoccus*, and *Caproiciproducens*. CST2-enriched genera (red) included *Flavobacterium*, CAG-632, *Clostridium_AP*, *Marvinbryantia*, and CAG-41. Labeled points indicate genera with FDR < 0.1. **(B)** Joint analysis of abundance fold change and prevalence odds ratio between CST types. Each point represents a genus; point size indicates statistical significance (−log₁₀ FDR). CST1-enriched genera showed both higher abundance and higher prevalence (upper right quadrant), while CST2-enriched genera clustered in the lower left quadrant. **(C)** Contingency table summarizing the distribution of genera by abundance and prevalence patterns. A total of 172 genera were enriched in both abundance and prevalence in CST1, whereas 56 genera showed the same pattern in CST2. Only 2 genera exhibited discordant patterns (higher abundance in CST2 but higher prevalence in CST1). **(D)** Prevalence comparison of representative differential genera between CST1 (blue) and CST2 (red). Left panel shows CST2-enriched genera with higher prevalence in CST2, including UMGS599, UMGS1901, *Flavobacterium*, and *Anaerosporobacter*. Right panel shows CST1-enriched genera with near-universal detection in CST1, including *Bifidobacterium*, *Lactococcus*, *Anaerococcus*, and *Eubacterium*.

Differential abundance analysis (ALDEx2) uncovered a clear taxonomic dichotomy (Figure 3A). CST1 was characterized by a constellation of maturation-associated anaerobes, including *Bifidobacterium*, *Collinsella*, *Anaerococcus*, *Caproiciproducens*, and *Absicoccus*. Conversely, CST2 was defined by the enrichment of opportunistic or dysbiotic taxa such as *Flavobacterium*, CAG-632, *Clostridium_AP*, *Marvinbryantia*, and CAG-41.

All differential abundance analyses were performed on day-21 metagenomes (Figure 3B). CST1-enriched genera occupied the high-abundance/high-prevalence quadrant, with 172 genera showing consistent enrichment across both metrics, compared to only 56 in CST2 (Figure 3C). Notably, core CST1 taxa (e.g., *Bifidobacterium*, *Lactococcus*) approached near-universal detection (80–100%) within their cluster, whereas CST2 markers (e.g., *Flavobacterium*, UMGS599) were highly prevalent in CST2 but rare in CST1 (Figure 3D). This pattern suggests that CST1 and CST2 represent taxonomically distinct day-21 ecological states. Given the small sample size and high-dimensional feature space, the differential genus-level results should be interpreted as exploratory community-level signatures rather than as a validated taxonomic biomarker panel.

### 3.4 Functional profiling and exploratory taxon–function associations differ between CST1 and CST2

**Figure 4:**
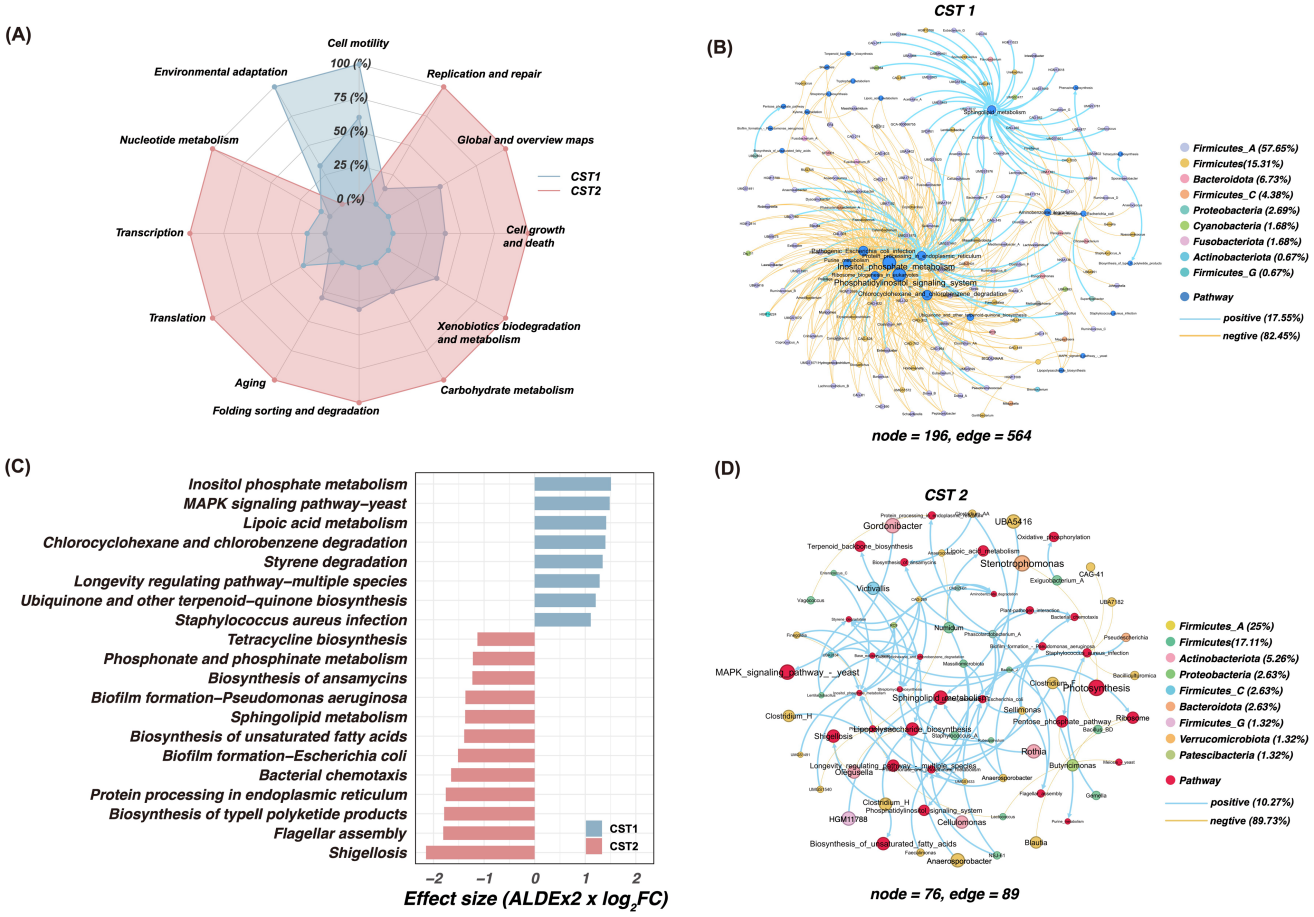
Functional profiling and exploratory taxon–function association maps differ between CST1 and CST2. **(A)** Radar plot comparing the relative abundance of KEGG pathway categories between CST1 (blue) and CST2 (red). CST1 showed higher proportions in replication and repair, nucleotide metabolism, and translation pathways, while CST2 was enriched in cell motility, xenobiotics biodegradation, and cell growth and death. **(B)** The network comprised 196 nodes and 564 edges and was used descriptively to visualize CST1-specific taxon–function co-variation. **(C)** Bar plot showing differentially abundant KEGG pathways between CST1 and CST2 (ALDEx2 analysis, FDR < 0.1). CST1 was enriched in pathways related to metabolic homeostasis, including inositol phosphate metabolism, lipoic acid metabolism, and ubiquinone biosynthesis. CST2 was enriched in pathways associated with motility and virulence, including flagellar assembly, bacterial chemotaxis, biofilm formation (*Escherichia coli* and *Pseudomonas aeruginosa*), and sphingolipid metabolism. **(D)** The CST2 association map comprised 76 nodes and 89 edges. These networks are presented as exploratory taxon–function association maps and should not be interpreted as validated ecological interaction networks.

Based on day-21 functional profiles (*n* = 23), CST1 and CST2 showed distinct metabolic and ecological signatures. CST1 was enriched in housekeeping pathways—replication, repair, nucleotide metabolism, and translation—while CST2 favored cell motility, xenobiotic degradation, and cell death (Figure 4A).

Pathway-level analysis (ALDEx2) confirmed this contrast (Figure 4C). CST1 showed enrichment in metabolic homeostasis pathways, including inositol phosphate and lipoic acid metabolism, ubiquinone biosynthesis, and longevity-regulating functions. In contrast, CST2 was enriched in virulence-related pathways such as flagellar assembly, chemotaxis, biofilm formation (*E. coli* and *P. aeruginosa*), and sphingolipid metabolism (FDR < 0.1), indicating higher motility and invasiveness.

Exploratory taxon–function association maps showed different co-variation patterns between CSTs. CST1 formed a denser taxon–pathway association map (196 nodes, 564 edges), whereas CST2 showed fewer nodes and edges (76 nodes, 89 edges) (Figures 4B,D). These network summaries were used descriptively to visualize CST-specific taxon–function co-variation. Because the within-CST sample sizes were small and the analysis was based on relative-abundance data, these patterns should not be interpreted as validated ecological interactions or as evidence of stable community organization or causal functional buffering.

### 3.5 Exploratory longitudinal changes from day 21 to week 36

We next used the paired day-21 and week-36 metagenomes to explore within-infant ecological changes over time. Across paired samples, Shannon diversity increased from day 21 to week 36 [median 1.324 to 1.651, paired Wilcoxon *p* = 0.002], and Simpson diversity also increased [median 0.481 to 0.629, *p* = 0.006]. Observed species richness and Chao1 richness showed non-significant increasing trends (both *p* = 0.093) (Supplementary Figure S4A and Supplementary Table S3). However, the Bray–Curtis distance between day 21 and week 36 did not differ significantly between infants initially assigned to CST1 and CST2 (*p* = 0.408; Supplementary Figure S4B and Supplementary Table S3), suggesting that this small, paired dataset does not provide strong evidence for CST-specific longitudinal trajectory differences.

### 3.6 Host–microbiome interactions correlate with inflammatory markers

**Figure 5:**
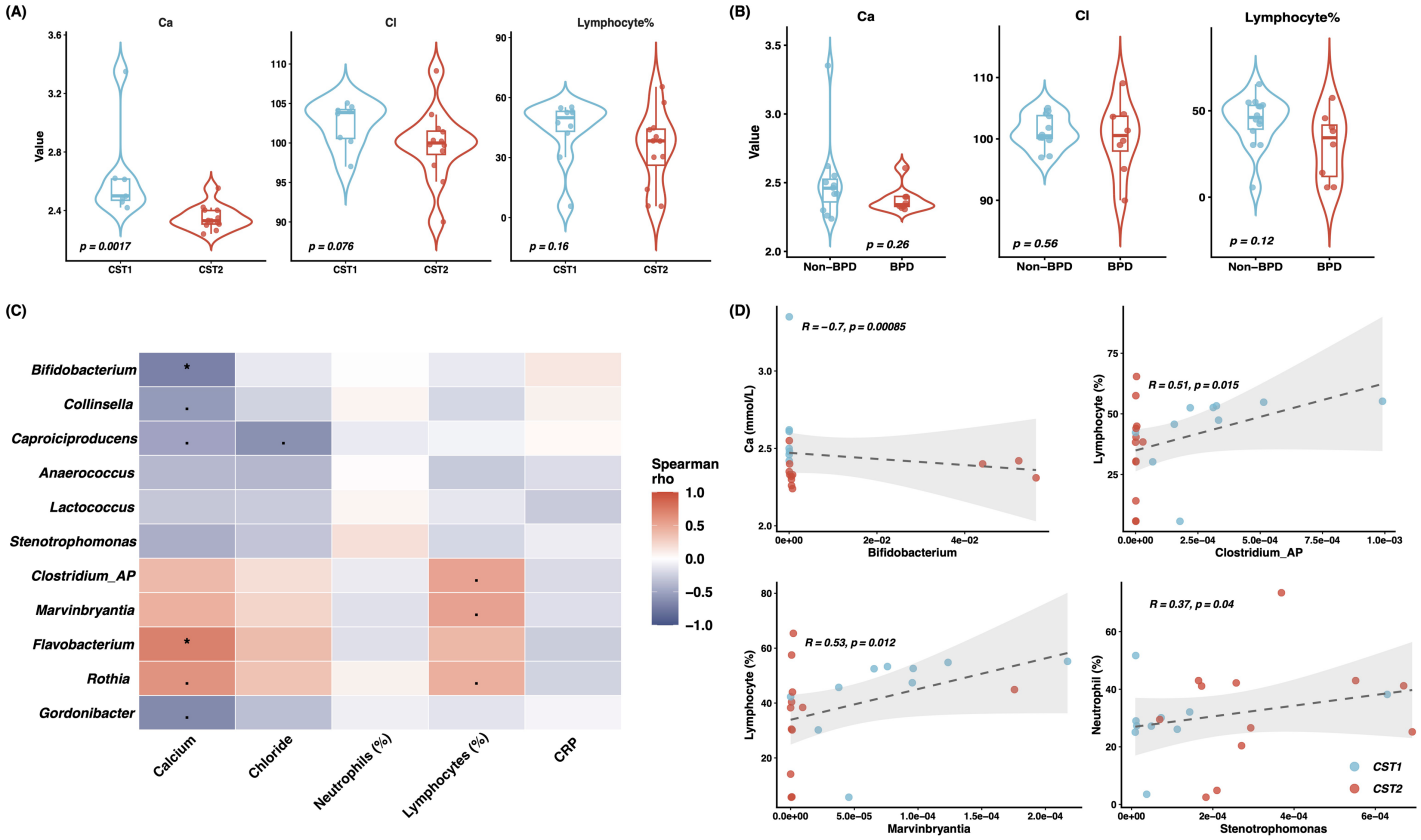
Associations between gut microbiota and host clinical parameters. **(A)** Comparison of serum calcium (Ca), chloride (Cl), and lymphocyte percentage between CST1 and CST2 at postnatal day 21. Serum calcium was significantly lower in CST2 compared to CST1 (*p* = 0.0017), while chloride showed a trend toward lower levels in CST2 (*p* = 0.076). **(B)** Comparison of the same clinical parameters between BPD and non-BPD groups. No significant differences were observed between groups. **(C)** Heatmap showing Spearman correlations between representative CST-associated genera and day-21 clinical markers. Benjamini–Hochberg correction was applied across all displayed correlations. Asterisks indicate FDR < 0.05, and plus signs indicate nominal *p* < 0.05 that did not survive FDR correction. **(D)** Representative scatter plots illustrating selected genus–clinical marker associations. The *Bifidobacterium*–calcium association remained significant after FDR correction and was further assessed using gestational-age-adjusted partial Spearman analysis. Statistical comparisons were performed using Mann–Whitney *U* tests **(A,B)** and Spearman correlation with Benjamini–Hochberg correction **(C,D)**. *FDR < 0.05; + nominal *p* < 0.05 only.

Clinical parameters were collected at day 21 to match the microbiome samples used in CST classification. While no significant differences were observed between BPD and non-BPD groups (Figure 5B), CST-based comparisons revealed lower serum calcium in CST2 infants (*p* = 0.0017) and a trend toward reduced chloride (*p* = 0.076); lymphocyte percentage did not differ (*p* = 0.16) (Figure 5A). Correlation analysis provided finer resolution (Figures 5C,D).

After Benjamini–Hochberg correction across genus–clinical marker correlations, only the associations of Bifidobacterium and Flavobacterium with serum calcium remained significant at FDR < 0.05. Bifidobacterium was negatively correlated with calcium (Spearman rho = −0.70, FDR = 0.023), whereas Flavobacterium was positively correlated with calcium (rho = 0.71, FDR = 0.023). Several additional genus–host marker associations, including correlations involving lymphocyte percentage and neutrophil percentage, were nominally significant but did not survive FDR correction and were therefore interpreted as exploratory signals only (Figure 5C and Supplementary Table S4). Because gestational age may confound both microbial colonization and electrolyte status, we further performed partial Spearman correlation controlling for gestational age. The negative association between Bifidobacterium and calcium persisted after adjustment (partial rho = −0.61, *p* = 0.005; Supplementary Figure S5 and Supplementary Table S4), although residual confounding cannot be excluded.

## 4 Discussion

In this small exploratory cohort, day-21 gut microbiota community state types (CSTs) were associated with formal BPD classification at 36 weeks postmenstrual age. BPD was observed more frequently among infants assigned to CST2 than among those assigned to CST1; however, the modest sample size, the limited number of BPD events, and the marked imbalance in gestational age preclude conclusions about independent prediction, causality, or clinical utility. The apparent discrimination of the CST-plus-gestational-age model was lower after leave-one-out cross-validation, indicating optimism in the apparent AUC estimate. Therefore, the present findings should be interpreted as preliminary evidence that day-21 CSTs capture ecological variation associated with BPD classification, rather than as validation of a clinical prediction tool. This cautious interpretation is consistent with prior pilot and multi-omics studies linking early gut microbiota alterations to BPD-related phenotypes while emphasizing the need for larger longitudinal validation cohorts (Chen et al., 2021; Ryan et al., 2019). It is also aligned with broader evidence that BPD prediction remains strongly dependent on clinical immaturity markers such as gestational age and respiratory support exposure (Jensen et al., 2019; Peng et al., 2022).

The taxonomic and functional signatures differentiating CST1 and CST2 suggest two contrasting day-21 ecological profiles. CST1 was enriched in Bifidobacterium and other commensal-associated taxa, whereas CST2 contained higher relative abundances of opportunistic taxa and pathways related to motility, chemotaxis, and biofilm-associated functions. These patterns are biologically plausible in the context of preterm gut development, where microbial succession is shaped by immaturity, antibiotic exposure, feeding progression, and NICU environmental pressures (Healy et al., 2022; Thänert et al., 2024). In particular, antibiotic exposure has been shown to disrupt microbiome maturation and prevent acquisition of beneficial metabolic functions in preterm infants, providing an important alternative explanation for dysbiotic community configurations such as CST2 (Xu et al., 2022). However, the feature-level findings in the present cohort were derived from high-dimensional analyses in a small sample and should therefore be interpreted as exploratory community-level signatures rather than as a validated taxonomic biomarker panel.

The alpha-diversity analyses provide additional ecological context for these CST differences. CST2 showed lower Shannon diversity, Simpson diversity, observed species richness, and Chao1 richness than CST1, while BPD infants showed reduced richness compared with non-BPD infants. Reduced diversity and delayed community maturation have frequently been reported in preterm gut microbiome studies, although their clinical meaning depends heavily on postnatal exposures and developmental stage (Groer et al., 2014; Pammi et al., 2017). Feeding is particularly relevant in this setting, because human milk, donor milk, and formula can differentially shape Bifidobacterium abundance and broader microbiome structure in preterm infants (Parra-Llorca et al., 2018). In our cohort, quantitative feeding-mode proportions at day 21 were unavailable for adjustment, so Bifidobacterium enrichment in CST1 cannot be interpreted as an exposure-independent microbial phenotype.

From a mechanistic standpoint, several pathways reported in prior studies may provide biological context for the CST-associated patterns observed here, but these mechanisms were not directly tested in the present cohort. Opportunistic taxa enriched in CST2 may be consistent with increased exposure to microbial products such as lipopolysaccharide or flagellin, which have been linked to innate immune activation along the gut–lung axis (Dang and Marsland, 2019). The gut microbiota is known to shape neonatal immune maturation, barrier function, and systemic inflammatory tone, all of which are relevant to lung development in preterm infants (Sanidad and Zeng, 2020). Conversely, Bifidobacterium-enriched communities may reflect a more mature commensal-associated state and have been linked in prior studies to immune education and reduced inflammatory tone in early life (Henrick et al., 2021). Nevertheless, the directionality of these associations remains unresolved. CST2 may reflect a downstream consequence of prematurity severity, respiratory support, delayed feeding progression, systemic inflammation, or other intensive-care exposures rather than an upstream contributor to BPD.

The taxon–function networks should also be interpreted cautiously. Although CST1 showed denser taxon–pathway co-variation than CST2, these analyses were based on small within-CST sample sizes and relative-abundance data. Correlation-based microbiome networks are sensitive to compositionality and sampling variation, and network topology alone cannot establish ecological resilience, stability, or causal microbial interactions (Coyte et al., 2015). Therefore, the networks in this study are best viewed as descriptive association maps that visualize CST-specific taxon–function co-variation. Future studies with larger sample sizes, longitudinal sampling, and compositionality-aware network methods will be needed to determine whether these patterns represent reproducible ecological organization.

**Figure 6:**
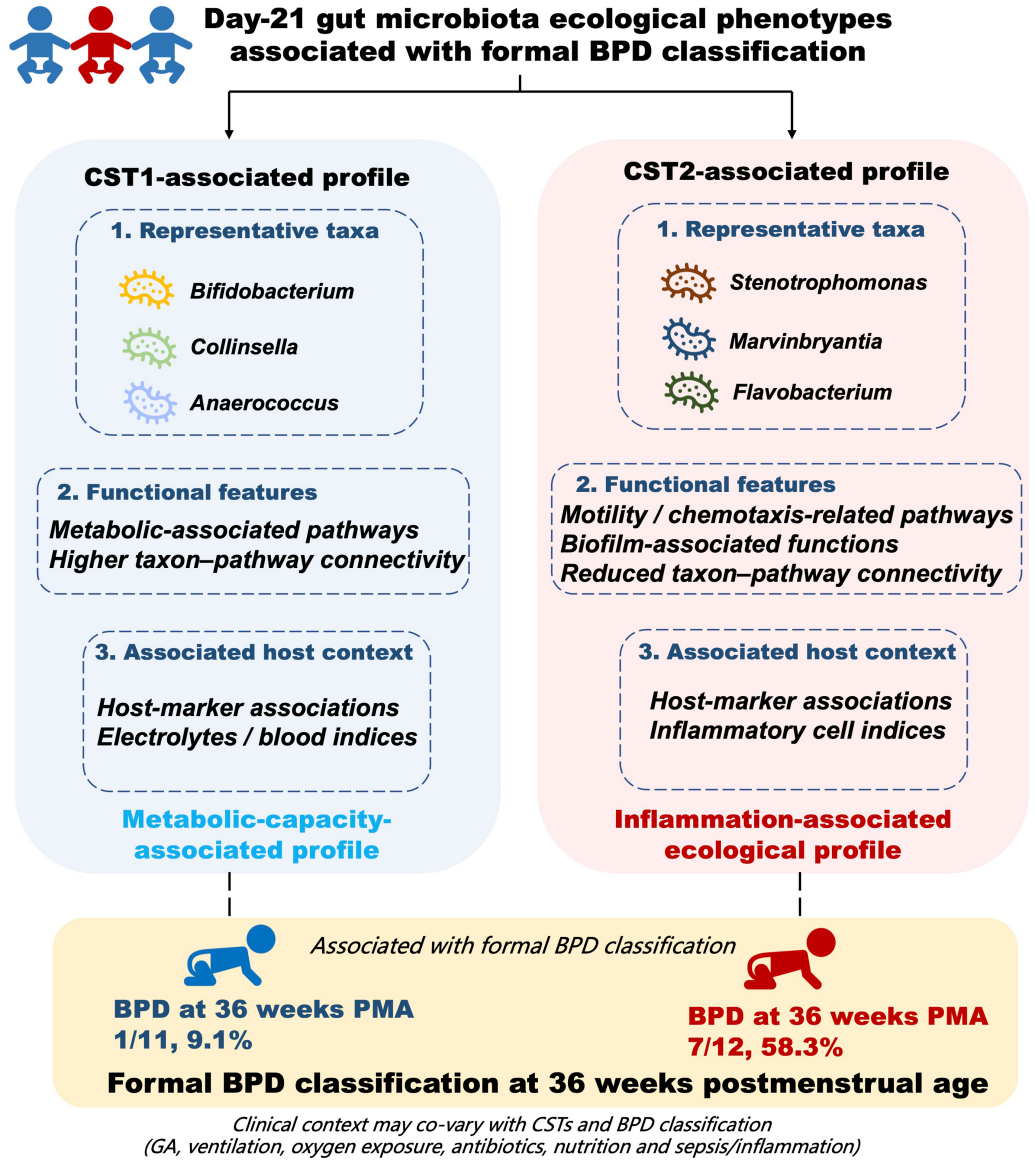
Conceptual summary of day-21 gut microbiota ecological phenotypes associated with formal BPD classification in preterm infants. Conceptual summary of day-21 gut microbiota ecological phenotypes associated with formal BPD classification in preterm infants. BPD was diagnosed in 9.1% of CST1 infants (1/11) and 58.3% of CST2 infants (7/12). This framework is associative and hypothesis-generating; it does not establish that CSTs causally contribute to BPD. Clinical factors including gestational age, birth weight, respiratory support, antibiotic exposure, feeding mode, probiotic use, and systemic inflammation may co-vary with both CST assignment and BPD classification.

We summarize the observed associations between day-21 CSTs, microbial features, host-marker correlations, and formal BPD classification in a conceptual framework (Figure 6). This framework is associative and hypothesis-generating. It illustrates how CST1 and CST2 differed in representative taxa, functional profiles, taxon–function co-variation, and BPD proportions, while explicitly acknowledging that clinical factors such as gestational age, birth weight, respiratory support, antibiotic exposure, feeding mode, probiotic use, and systemic inflammation may co-vary with both CST assignment and BPD classification. This interpretation is consistent with recent work showing that microbiome development in hospitalized preterm infants is strongly shaped by both antibiotic and non-antibiotic NICU exposures (Thänert et al., 2024). Whether day-21 ecological differences actively shape respiratory trajectories or instead reflect the cumulative impact of prematurity and clinical care cannot be resolved from these data alone.

The paired day-21 and week-36 data provide a preliminary view of within-infant ecological change over time. Shannon and Simpson diversity increased from day 21 to week 36, consistent with progressive postnatal microbial maturation in preterm infants. However, the Bray–Curtis distance between day 21 and week 36 did not differ significantly between infants initially assigned to CST1 and CST2, suggesting that this small paired dataset does not provide strong evidence for CST-specific longitudinal trajectory differences. Prior longitudinal studies have shown that repeated sampling is essential for determining whether microbiome alterations precede, accompany, or follow respiratory disease progression in preterm infants (Ryan et al., 2019; Thänert et al., 2024). Future studies should therefore integrate serial microbiome profiling with time-varying clinical exposures, rather than relying on a single day-21 sampling point.

Several limitations are central to the interpretation of this study. First, the cohort was small, with only eight BPD events, which limits statistical power, increases the risk of false-positive feature-level findings, and precludes stable multivariable prediction. Second, gestational age differed substantially between CST groups and between BPD and non-BPD infants, indicating that prematurity severity is an important confounder of the CST–BPD association. Third, although no systemic antibiotics were administered within 14 days before day-21 stool sampling and no infants received probiotic supplementation, detailed lifetime antibiotic exposure, sepsis episodes, and quantitative feeding-mode proportions were unavailable for formal adjustment. Fourth, the maturation-distance analysis used an internal week-36 non-BPD reference and should therefore be viewed as corroborative rather than independent evidence of delayed maturation. Fifth, the bacterial metagenomic analysis did not capture fungal or viral communities, although fungal dysbiosis has also been implicated in BPD-related microbial ecology (Willis et al., 2023). Finally, the relatively later gestational age of this cohort may limit generalizability to extremely preterm infants at highest risk for severe BPD.

Future studies may test whether CST-informed stratification can guide microbiome-focused monitoring or intervention trials. However, the present observational pilot study does not provide sufficient evidence to recommend specific probiotic, postbiotic, or clinical interventions. This caution is important because probiotic-induced microbiome changes do not necessarily translate into improved clinical endpoints, as illustrated by the PRIMAL randomized clinical trial of multistrain Bifidobacterium and Lactobacillus supplementation in preterm infants (Van Rossum et al., 2024). Longitudinal metagenomic tracking combined with appropriately powered, prospectively designed studies will be essential to determine whether microbiota modulation can shift ecological trajectories and whether such shifts translate into meaningful reductions in BPD incidence or severity.

## Data Availability

The processed and de-identified metagenomic abundance tables, community state type assignments, and analysis scripts generated and analyzed during this study are publicly available at GitHub: https://github.com/WenjunXiongBear/BPD-day21-gut-metagenomics.
