# Supplementary material for: Day-21 gut microbiota community state types are associated with bronchopulmonary dysplasia classification in preterm infants: a pilot shotgun metagenomic study
Source: Front Microbiol. 2026 Jul 6;17:1835952. doi: 10.3389/fmicb.2026.1835952 (PMC13381461; doi:10.3389/fmicb.2026.1835952)
Supplement: Supplementary file 4 [file Table_1.DOCX]

**Fig. S1. Alpha diversity differences by day-21 CST and BPD status.**

(A) Alpha diversity metrics compared between CST1 and CST2 at day 21, including Shannon diversity, Simpson diversity, Chao1 richness, and observed species richness. CST2 showed significantly lower alpha diversity than CST1 across all four metrics.

(B) Alpha diversity metrics compared between non-BPD and BPD infants at day 21. BPD infants showed significantly lower Chao1 richness and observed species richness, whereas Shannon and Simpson diversity showed non-significant downward trends.

Box plots show median and interquartile range; points represent individual infants. Between-group comparisons were performed using Wilcoxon rank-sum tests. *p < 0.05, **p < 0.01, ns, not significant.

**Fig. S2. Sequencing-depth metrics and rarefaction curves.**

(A) Sequencing-depth and read-processing metrics compared between CST1 and CST2 at day 21, including raw reads, clean reads, host-removed reads, host rate, and Q30. None of these metrics differed significantly between CST groups.

(B) Rarefaction curves based on species-level absolute abundance profiles from day-21 metagenomes, showing expected species richness across rarefied read counts.

Box plots show median and interquartile range; points represent individual infants. Between-group comparisons were performed using Wilcoxon rank-sum tests. ns, not significant.

**Fig. S3. Gestational-age imbalance and exploratory ROC analysis.**

(A) Gestational age compared between CST1 and CST2 infants at day 21.

(B) Gestational age compared between non-BPD and BPD infants.

(C) Apparent and LOOCV AUCs are shown for the CST-plus-gestational-age model.

Box plots show median and interquartile range; points represent individual infants. Between-group comparisons were performed using Wilcoxon rank-sum tests. ROC curves are shown for exploratory discrimination only and should not be interpreted as evidence of clinical predictive utility.

**Fig. S4. Within-infant microbiome changes from day 21 to week 36.**

(A) Paired alpha-diversity trajectories from day 21 to week 36, including Shannon diversity, Simpson diversity, Chao1 richness, and observed species richness. Lines connect paired samples from the same infant.

(B) Bray–Curtis distance between paired day-21 and week-36 samples compared by day-21 CST assignment.

Paired day-21 to week-36 alpha-diversity comparisons were performed using paired Wilcoxon tests. Between-CST comparisons of Bray–Curtis trajectory distance were performed using Wilcoxon rank-sum tests. ns, not significant.

**Fig. S5. Bifidobacterium–calcium association after adjustment for gestational age.**

Scatter plot showing the association between Bifidobacterium relative abundance and serum calcium at day 21. The unadjusted Spearman correlation was rho = −0.70 (p < 0.001). Partial Spearman correlation controlling for gestational age showed that the association remained present after adjustment (partial rho = −0.61, p = 0.005). The fitted line is shown for visualization only.
