# Supplementary material for: Day-21 gut microbiota community state types are associated with bronchopulmonary dysplasia classification in preterm infants: a pilot shotgun metagenomic study
Source: Front Microbiol. 2026 Jul 6;17:1835952. doi: 10.3389/fmicb.2026.1835952 (PMC13381461; doi:10.3389/fmicb.2026.1835952)
Supplement: Supplementary file 5 [file Table_2.DOCX]

**Table S2. Exploratory logistic regression outputs.**

This table summarizes exploratory logistic regression models assessing the association of CST2 status and gestational age with BPD classification.

***Exploratory logistic regression models for BPD (n = 18, events = 8).***

| **Model** | **Term** | **β** | **SE** | **z** | **P value** | **OR (95% CI)** |
| --- | --- | --- | --- | --- | --- | --- |
| **Model 1: CST2 only** | *(Intercept)* | -2.303 | 1.049 | -2.20 | 0.028 | — |
|  | CST2 | 2.639 | 1.201 | 2.20 | 0.028 | 14.00 (1.330–147.38) |
| **Model 2: GA only** | *(Intercept)* | 22.326 | 9.497 | 2.35 | 0.019 | — |
|  | GA (wk) | -0.781 | 0.325 | -2.40 | 0.016 | 0.458 (0.242–0.866) |
| **Model 3: CST2 + GA** | *(Intercept)* | 18.536 | 10.677 | 1.74 | 0.083 | — |
|  | CST2 | 1.075 | 1.397 | 0.77 | 0.442 | 2.929 (0.190–45.24) |
|  | GA (wk) | -0.676 | 0.353 | -1.91 | 0.056 | 0.509 (0.255–1.016) |

*Abbreviations:* BPD, bronchopulmonary dysplasia; CI, confidence interval; CST, community state type; GA, gestational age; OR, odds ratio; SE, standard error; wk, weeks. *Notes:* Logistic regression models were fitted with BPD (yes/no) as the outcome. Model 1 included CST2 (vs CST1) as the sole predictor; Model 2 included GA in completed weeks as the sole predictor; Model 3 included both CST2 and GA. Coefficients (β), standard errors, z statistics, and Wald P values are shown alongside ORs with profile-likelihood 95% CIs. Intercept ORs are not biologically interpretable in this setting and are denoted by an em-dash. Given the small sample size and limited events-per-variable, results are exploratory; the CI for CST2 in Model 1 is wide, and the CST2 effect attenuates substantially after adjustment for GA, consistent with collinearity between CST and gestational maturity rather than an independent CST effect. For consistency across exploratory models, logistic regression analyses were fitted using the same complete-case subset available for the CST2-plus-gestational-age model; therefore, the model sample size was n = 18.
